# Genome-Wide Association Study of Opioid Cessation

**DOI:** 10.3390/jcm9010180

**Published:** 2020-01-09

**Authors:** Jiayi W. Cox, Richard M. Sherva, Kathryn L. Lunetta, Emma C. Johnson, Nicholas G. Martin, Louisa Degenhardt, Arpana Agrawal, Elliot C. Nelson, Henry R. Kranzler, Joel Gelernter, Lindsay A. Farrer

**Affiliations:** 1 Department of Medicine (Biomedical Genetics), Boston University School of Medicine, Boston, MA 02118, USA; jiayiwu@bu.edu (J.W.C.); sherva@bu.edu (R.M.S.); 2 Department of Biostatistics, Boston University School of Public Health, Boston, MA 02118, USA; klunetta@bu.edu; 3 Department of Psychiatry, Washington University in St. Louis, St. Louis, MO 63130, USA; emma.c.johnson@wustl.edu (E.C.J.); arpana@wustl.edu (A.A.); nelsone@psychiatry.wustl.edu (E.C.N.); 4 School of Psychology, The University of Queensland, St Lucia QLD 4072, Australia; Nick.Martin@qimrberghofer.edu.au; 5 School of Medicine, University of New South Wales, Sydney NSW 2052, Australia; l.degenhardt@unsw.edu.au; 6 Perelman School of Medicine, University of Pennsylvania and VISN 4 MIRECC, Crescenz VAMC, Philadelphia, PA 19104, USA; kranzler@pennmedicine.upenn.edu; 7 Departments of Psychiatry, Genetics and Neuroscience, Yale School of Medicine, New Haven, CT 06511, USA; joel.gelernter@yale.edu; 8 Department of Psychiatry, VA CT Healthcare Center, West Haven, CT 06516, USA; 9 Departments of Neurology, Ophthalmology and Epidemiology, Boston University Schools of Medicine and Public Health, Boston, MA 02118, USA

**Keywords:** genome-wide association study, opioid cessation, opioid use disorder, shared genetic risk, polygenic risk score

## Abstract

The United States is experiencing an epidemic of opioid use disorder (OUD) and overdose-related deaths. However, the genetic basis for the ability to discontinue opioid use has not been investigated. We performed a genome-wide association study (GWAS) of opioid cessation (defined as abstinence from illicit opioids for >1 year or <6 months before the interview date) in 1130 African American (AA) and 2919 European ancestry (EA) participants recruited for genetic studies of substance use disorders and who met lifetime Diagnostic and Statistical Manual of Mental Disorders, 5th Edition (DSM-5) criteria for OUD. Association tests performed separately within each ethnic group were combined by meta-analysis with results obtained from the Comorbidity and Trauma Study. Although there were no genome-wide significant associations, we found suggestive associations with nine independent loci, including three which are biologically relevant: rs4740988 in *PTPRD* (*p*_AA + EA_ = 2.24 × 10^−6^), rs36098404 in *MYOM2* (*p*_EA_ = 2.24 × 10^−6^), and rs592026 in *SNAP25-AS1* (*p*_EA_ = 6.53 × 10^−6^). Significant pathways identified in persons of European ancestry (EA) are related to vitamin D metabolism (*p* = 3.79 × 10^−2^) and fibroblast growth factor (FGF) signaling (*p* = 2.39 × 10^−2^). UK Biobank traits including smoking and drinking cessation and chronic back pain were significantly associated with opioid cessation using GWAS-derived polygenic risk scores. These results provide evidence for genetic influences on opioid cessation, suggest genetic overlap with other relevant traits, and may indicate potential novel therapeutic targets for OUD.

## 1. Introduction

Non-prescribed use of opioid analgesics has become a significant global problem that affects the health and economic welfare of society [1]. In 2015, more than 33,000 Americans died of an opioid overdose, and about half of these deaths involved prescription opioids [2]. The US Department of Health and Human Services declared a public health emergency in 2017 to address the national opioid crisis [3]. Opioid maintenance and cognitive behavioral approaches are effective [4,5], but they have limited long-term value as reflected in high dropout and relapse rates [6]. Nearly 80% of Americans with opioid use disorder (OUD) do not seek treatment [7], which adds to the burden of this devastating public health problem. Family and twin studies have shown that OUD is moderately heritable (*h*^2^ = 0.43–0.50) [8]. Many gene loci for OUD have been identified by linkage [9,10,11], candidate gene [12,13,14,15], and genome-wide association study (GWAS) [16,17,18,19] approaches. The lack of published reports about genetic factors that influence successful cessation of illicit opioids and prescription opioid misuse may be due to the challenge of assembling sufficiently large cohorts with the requisite information about opioid use and cessation, high relapse rate, and non-standard definitions of cessation [20,21].

Here, we present results from the first genome-wide association study (GWAS) of opioid cessation among persons meeting the Diagnostic and Statistical Manual of Mental Disorders, 5th Edition (DSM-5) criteria for OUD [22] in a sample of African Americans (AAs) and persons of European ancestry (EAs) recruited for genetic studies of substance use disorders (SUDs) [16,17,18,19,23]. We also identified genetic overlap between opioid cessation and other SUD-related traits in the UK Biobank (UKBB) [24].

## 2. Materials and Methods

### 2.1. Participants and Diagnostic Procedures

Study participants were ascertained from two sources. The Yale–Penn sample includes 6188 AA and 6835 EA participants with and without substance use disorders who were enrolled in genetic studies of dependence on opioids, cocaine, or alcohol between 2000 and 2017 through treatment clinics at the University of Connecticut Health Center, Yale University School of Medicine, the Medical University of South Carolina, University of Pennsylvania, and McLean Hospital in Belmont, Massachusetts [25,26]. This cohort included affected sibling pairs and additional family members, as well as unrelated cases and controls. Probands with a diagnosis of schizophrenia or bipolar disorder were excluded [25,26]. A computerized version of the Semi-Structured Assessment for Drug Dependence and Alcoholism (SSADDA) [27,28] was administered to all participants and diagnoses of dependence on various substances and other psychiatric disorders were derived according to DSM criteria. A second sample of European-origin Australians was derived from the Comorbidity and Trauma Study (CATS) which has been described previously [29]. In brief, opioid-dependent (OD) cases ages 18 or older were recruited from opioid substitution therapy (OST) clinics in metropolitan Sydney, Australia. Persons who had recent suicidal intent or psychosis were excluded. Controls were recruited from neighborhoods geographically proximal to the OSTs and excluded persons who used opioids recreationally more than five times (i.e., only OD cases were included in this analysis). Participants were interviewed using the Semi-Structured Assessment for the Genetics of Alcoholism-Australia (SSAGA-OZ) [30], which was used to derive DSM-IV SUD diagnoses. Institutional review board approval for this study was obtained at all participating institutions, and written informed consent was obtained from all study participants. 

### 2.2. Cessation Phenotype Definition

Participants who were eligible for this analysis endorsed at least two DSM-5 OUD criteria, corresponding to a lifetime diagnosis of DSM-5 OUD. Current opioid cessation status was determined based on the response to the SSADDA question, “When was the last time you used an (illicit) opioid drug (including illicit methadone)”. We classified individuals who last used an opioid >1 year before the date of interview as having successfully ceased opioid use and those whose last use of an opioid was <6 months before the interview date as having not ceased opioid use. Opioid users whose self-reported last use was 6–12 months before were excluded from the analysis, thus yielding a sample of 1130 AAs and 1859 EAs for the opioid cessation study. The ascertainment scheme and filtering steps are shown in Supplemental Figure S1. Persons in the CATS dataset with a lifetime diagnosis of OD were classified as ceased if their last use of an opioid was at least one year before the age at the interview (*n* = 337) or non-ceased if the age of last use of an opioid was the same as the age at the interview (*n* = 723). OD cases in the CATS sample who last used an opioid exactly one year before the last interview were excluded. Characteristics of all individuals included in the GWAS are shown in Table 1. 

### 2.3. Genotyping, Imputation, and Quality Control

DNA specimens in the Yale–Penn sample were genotyped using the Illumina HumanOmni1-Quad v1.0 microarray (OMNI) (Illumina, Inc., San Diego, USA), which contains 988,306 autosomal single nucleotide polymorphisms (SNPs); the Illumina Infinium Human Core Exome microarray (HCE), which contains 265,919 exome-focused SNPs and approximately 240,000 tagging SNPs to allow genome-wide imputation; or the Illumina Multi-ethnic Global Array (MEGA), which contains 1,779,819 markers with appreciable frequency in at least one of five major populations to maximize genome-wide imputation accuracy. Genotyping of the Yale–Penn sample was performed at the Yale Center for Genome Analysis or the Gelernter Lab except for a group of participants (822 AAs and 955 EAs) that was genotyped using the OMNI1 array at the Center for Inherited Disease Research (CIDR). DNA specimens from the CATS sample were genotyped at CIDR using the Illumina Human660W-Quad BeadChip.

Quality control of genotype data was performed as previously described [8]. Briefly, individuals with a call rate <98% and variants with minor allele frequency (MAF) <1% were excluded. Pairwise identity-by-decent (IBD) was calculated with PLINK [31] to determine genetic relatedness among individuals in the sample. Individuals with a pairwise IBD estimate >25% were assigned to the same family. Self-reported males with X chromosome heterozygosity >20% and self-reported females with X chromosome heterozygosity <20% were excluded. Population substructure in the entire sample was evaluated by analysis of principal components (PC) of ancestry using Eigensoft [32] and the multi ethnic 1000 Genome reference panel for comparison. Individuals were classified as AA or EA according to the reference panel population that they more closely matched. SNP genotype imputation was performed separately in AAs and EAs using the March 2012 1000 Genomes reference panel (1000 Genomes Project, 2012; http://www.1000genomes.org/) and IMPUTE2 [33] implemented on the University of Michigan imputation server (https://imputationserver.sph.umich.edu).

### 2.4. Genetic Association Analysis

Association of opioid cessation with dosage of the minor allele of each SNP was evaluated using logistic regression models solved with generalized estimating equations (GEE) to correct for correlations among related individuals and included terms for age, sex, and the first five PCs to correct for population sub-structure. Association testing was restricted to SNPs with an imputation score >0.8 and MAF >3%. Association tests were performed separately within each population and within each genotyping platform to account for batch effects. To prevent low-frequency SNPs from inflating test statistics, a filter based on the effective number of alleles in cases was used, and SNPs with fewer than 10 effective alleles (*N*_eff_ = 2 × number achieving successful cessation × imputation quality × MAF) were excluded. A cutoff of 10 was chosen to control for type I error. For the CATS dataset, we obtained summary statistics of SNPs with MAF > 0.01 and imputation R^2^ > 0.3 and, logistic association was performed using PLINK v.1.9 [34] adjusting for sex, age, and the first three PCs (determined by examining the number of PCs associated with opioid cessation in the dataset). Results were corrected for genomic inflation (λ) and combined across population and batch groups via inverse variance weighted meta-analysis implemented in the program METAL [35]. The genome-wide significance (GWS) threshold was set at *p* < 5.0 × 10^−8^. A threshold of *p* < 1.0 × 10^−5^ was used to define suggestive association.

#### Power

Power to detect a significant association with opioid cessation in the study sample was evaluated separately for AAs and EAs using a genetic power calculator [36], assuming α = 5 × 10^−8^, power (1 − β) = 0.8 and an additive model. These analyses indicated sufficient power to detect a variant with an MAF of ≥0.04 and heterozygous genotype relative risks of 2.00 in AAs and 1.62 in EAs, or a variant with an MAF of 0.1 and heterozygous genotype relative risks of 1.70 in AAs and 1.40 in EAs. 

### 2.5. Assessment of SNP Effects on Gene Expression

SNPs that surpassed a threshold of *p* < 1 × 10^−5^ in the opioid cessation GWAS were assessed for their potential to affect gene expression using information in the Genotype Tissue Expression Portal (GTEx) (http://www.gtexportal.org) [37] and Braineac (http://www.braineac.org/) [38] databases. GTEx contains information that links SNP genotype to expression in multiple human tissues, whereas Braineac incorporates expression data for multiple brain regions derived from 130 individuals that were obtained from the UK Brain Expression Consortium (UKBEC) and contains information on SNPs that can affect gene expression for multiple brain regions. 

### 2.6. Analysis of Genetic Overlap with Other Traits

Genetic overlap between opioid cessation and several OUD-related traits was also evaluated in the UKBB [39]. Relevant phenotypes in the UKBB included “former drinker” or “ex-smoker” which reflects an ability to cease use of other substances, and “back pain for more than three months”. “Back pain for more than three months” was chosen instead of other pain-related traits because back pain is the most common form of pain and also a leading cause of disability [40,41]. We used PRSice [42] to calculate polygenic risk scores (PRSs) based on summary statistics for these traits to estimate their association with opioid cessation. These analyses were conducted for EAs only because the majority of participants in the UKBB are EAs. PRSice identifies the optimal *p*-value threshold that explains the highest proportion of the trait variance for constructing PRSs. PRSice models assume an additive effect for each SNP, and SNPs in high linkage disequilibrium (LD) (R^2^ > 0.25) were removed using 200 bp sliding windows [42]. PRSs were calculated by summing the product of the risk allele count reported in the UKBB GWAS from opioid cessation samples multiplied by the effect reported in the UKBB GWAS. Results were corrected for multiple testing using the Bonferroni method (*N*_AA_ = 3, *N*_EA_ = 6). 

### 2.7. Pathway Analysis

Population-specific GWAS summary statistics were assessed for enrichment of functionally related genes using MAGENTA [43]. MAGENTA combines individual variant association *p*-values into a gene score and computes a *p*-value that is corrected for gene size, the number of variants, and LD. A nominal *p*-value is then calculated for each gene set, defined as the fraction of randomly sampled gene sets of identical size less than either the 95th or 75th percentile of gene score *p*-values, after multiple testing correction. We used the 75th percentile cutoff for significance because it demonstrates greater power for highly polygenic traits with weak effect associations [43]. 

## 3. Results

### 3.1. Association of Demographic Factors with Opioid Cessation

In the Yale–Penn dataset, females had increased odds of cessation in both AAs (odds ratio [OR] = 1.33, *p* = 1.40 × 10^−2^) and EAs (OR = 1.27, *p* = 1.84 × 10^−3^). Older age was associated with increased odds of longer cessation in AAs (OR = 1.05 per year, *p* = 8.34 × 10^−12^) and EAs (OR = 1.05 per year, *p* = 2.00 × 10^−20^). In the CATS dataset, persons who ceased using opioids were 4.7 years older than those who did not (*p* = 4.49 × 10^−4^). Although individuals with mild OUD were included, the mean number of OUD criterion counts among participants in both datasets corresponds to a severe diagnosis. Additional characteristics of the study sample are shown in Table 1.

### 3.2. Genetic Association Findings for Opioid Cessation

There was no evidence of *p*-value inflation in either population group (Supplemental Figure S2). Although no SNPs reached the GWS level (Supplemental Figure S3), SNPs showing suggestive evidence of association (*p* < 1 × 10^−5^) with opioid cessation were located in nine independent regions (Table 2), including three that were specific to EAs (top SNP rs36098404 in a *MYOM2* intron: *p* = 4.11 × 10^−6^, OR = 1.72) and six in the combined sample (top SNP rs11767417 in a *SEPT14* intron: *p* = 1.30 × 10^−6^, OR = 1.39). Several of these associations were supported by evidence with surrounding SNPs in LD (Supplemental Figure S4).

### 3.3. Biological Pathways and Gene Sets Related to Opioid Cessation

After multiple test correction, significantly enriched pathways were observed in EAs for vitamin D metabolism (*p*_adj_ = 3.79 × 10^−2^) containing nine genes while only three were expected (Table S1A) and fibroblast growth factor (FGF) signaling (*p*_adj_ = 2.39 × 10^−2^) containing 20 genes while only 10 were expected (Table S1B). No pathways were significantly enriched in AAs.

### 3.4. Polygenic Overlap with Other SUDs and OUD-Related Ttraits

Table 3 shows that the PRS for back pain persistent for more than three months was significantly associated with cessation in EAs (*p* = 1.25 × 10^−7;^). Opioid cessation was also associated with PRSs for being a former drinker (*p* = 6.24 × 10^−4^) and former smoker (*p* = 4.44 × 10^−3^). However, the proportion of variance for opioid cessation explained by the trait PRSs were small, ranging from R^2^ = 0.0075 for being a former smoker to R^2^ = 0.021 for having back pain for more than three months in EAs.

## 4. Discussion

To our knowledge, this is the first GWAS of opioid cessation. Although no genome-wide significant associations were identified, we obtained suggestive evidence for association of cessation from opioid use for at least one year among persons who were diagnosed with OUD with variants in several genes not previously linked to OUD. Pathway analysis that was seeded with summary GWAS information implicated two biological processes in successful opioid abstinence, including vitamin D metabolism and FGF signaling. Analyses of PRSs computed for ability to quit smoking or drinking, and persistent back pain suggest a shared genetic underpinning of these traits with opioid cessation. 

Several of the top-ranked genes associated with opioid cessation have been implicated previously in substance use disorders or other psychiatric traits. Protein tyrosine phosphatase receptor type D (*PTPRD*) is abundantly expressed in central nervous system ventral midbrain neurons involved with reward, locomotor, and sleep processes in both mice and humans [44] and is an important regulator of axon growth [45]. Myomesin 2 (*MYOM2*) encodes a major protein in muscle tissue and was nominally associated with nicotine and alcohol dependence in Australian and Dutch populations [46]. Synaptosomal-associated protein of 25 kDa (*SNAP-25*) controls the release of neurotransmitters by modulating voltage-gated calcium channels [47]. Genes in the calcium channel signaling pathway are associated with OUD [16]. Studies have also linked *SNAP-25* to antipsychotic treatment response [48], morphine-associated contextual memory retrieval [49], and the risk of attention deficit hyperactivity disorder [50]. The top variant rs592026 (Supplemental Figure S4D) in *SNAP-25-AS1* is located 113 kb upstream of *SNAP-25* and is an expression quantitative trait locus for *SNAP-25* in thalamus (*p* = 1.8 × 10^−4^). 

A role in addiction-relevant biology for vitamin D metabolism and FGF signaling pathways identified from biological pathway analyses is supported by previous research. A neuroprotective effect of vitamin D against methamphetamine-induced dopamine depletion has been demonstrated [51]. Vitamin D3 has been shown to reduce neuropathic pain by modulating opioid signaling in the rodent brain [52]. A low level of vitamin D was observed in methadone maintenance patients [53] and associated with a higher dose opioid for treatment of cancer patients [54]. In addition, vitamin D inadequacy results in poorer physical functioning and health perception [55]. An explanation for this observation is that individuals who cease opioid use might be more physically fit due to better vitamin D metabolism than persistent opioid users. Several other genes in the vitamin D metabolic pathway have been linked to opioids. *CYP2D6* (*p*_best_SNP_ = 0.02) is a member of the cytochrome P450 family and encodes an enzyme that has many opioid substrates [56]. Polymorphisms in *CYP2D6* can dramatically affect the metabolic capacity leading to under- or over-exposure to opioids [57]. *CYP2D6* ultra-rapid metabolizers are more likely to experience adverse events with opioid treatment [58]. In the presence of opioids, *RARA* (*p*_best_SNP_ = 0.02) forms a complex with *RXRA*, which activates the mu-opioid receptor and modulates drug-seeking behaviors [59]. Some mutations in the vitamin D receptor gene (*VDR, p*_gene_ = 0.04, *p*_best_SNP_ = 0.001) have been associated with impulsivity in the context of alcohol dependence [60]. 

The FGF pathway has been previously implicated in opioid metabolism. One study showed that FGF is involved in the development of analgesic tolerance to the opioid agonist morphine [61]. In addition, the FGF receptor was identified as a converging site between mu-opioid receptor and growth factor signaling pathways [62]. One of the significantly enriched genes identified from this pathway, *MAP3K6* (*p*_gene_ = 0.09, *p*_best_SNP_ = 0.01), is differentially expressed in striatum and prefrontal cortex among mice treated acutely and chronically with morphine [63]. There are also links between vitamin D metabolism and FGF signaling. *FGF23*, a fibroblast growth factor that is highly expressed in bone, reduces levels of activated vitamin D [64] and thus leads to reduced bone mineral density and a higher risk of fracture [65]. Interestingly, long-term use of opioid analgesics has been associated with bone loss [66]. 

PRS analysis revealed genetic overlap between opioid cessation and several other traits. Although the observed associations of opioid cessation with PRS for former smokers and former drinking may be due to the ascertainment of our sample which is enriched for individuals dependent on multiple substances, shared genetic underpinnings between opioid cessation and risk of other SUDs is supported by studies in twins [67] and unrelated individuals [68]. Pathways enriched for opioid cessation are known to affect the direct downstream targets of cocaine and alcohol, for example, FGF signaling is responsible for the development and function of dopamine neurons [69], and vitamin D level regulates gamma-aminobutyric acid (GABA) release [70]. It is not surprising that chronic pain is genetically overlapped with greater substance use among patients on methadone treatment [71]. However, randomized clinical trials suggest that patients with chronic pain do not receive a benefit from opioid treatment, but suffer from more opioid-induced adverse events [40,72]. The analgesic effect of opioids might explain the positive association between chronic pain and opioid cessation. In addition, the Netrin-1 receptor gene, *DCC*, was related to chronic opioid exposure and chronic back pain in both mice and human studies [73,74]. 

Several limitations of our study should be noted. First, we used cross-sectional data to study a phenotype that would require long-term follow-up to define cessation more accurately. It is likely that some people who ceased opioids at the time of interview subsequently resumed opioid use. Second, we used a slightly different definition for cessation in the CATS dataset that was derived from reports of age at last use which resulted in a timeframe of last opioid use that differed from that of participants in the Yale−Penn dataset (e.g., CATS participants whose last use was 6–12 months prior to their interview were counted as not ceased, and ceased was defined more conservatively as last use greater than a year prior to interview). These differences might limit the utility of CATS as a replication dataset. Third, the opioid cessation GWAS sample had limited power to detect genome-wide significant association signals. For example, a sample of 300 additional cases would be needed to attain 80% of power to detect genome-wide significance for the top-ranked variant rs11767417. However, despite sub-genome-wide significant associations, we observed supportive evidence for association of the top variants with adjacent SNPs (Supplemental Figure S4). Future studies with adequately powered samples should be conducted to validate these findings. Finally, although small, the proportion of variance for opioid cessation explained by the significant trait PRS is comparable to that in similar studies [75].

## 5. Conclusions

Despite an absence of genome-wide significant variants for opioid cessation, these results provide evidence for genetic influences on opioid cessation, suggest genetic overlap with other relevant traits, and may indicate potential novel therapeutic targets for OUD. 

## Figures and Tables

**Table 1:** Characteristics of opioid-dependent persons in the Yale−Penn and Comorbidity and Trauma Study (CATS) datasets by cessation status.

	Yale–Penn ^a^						CATS ^b^		
	African Ancestry			European Ancestry			European Ancestry		
Cessation Status	Not Ceased	Ceased	Not Classified	Not Ceased	Ceased	Not Classified	Not Ceased	Ceased	Not Classified
Total *n* (% female)	682 (32.3)	448 (40.6)	101 (30.7)	1235 (33.3)	624 (41.3)	344 (37.5)	723 (37.1)	337 (41.8)	166 (48.2)
Age (SD) in years	41.5 (8.4)	45.2 (8.4)	43.7 (9.1)	30.1 (10.2)	40.3 10.7)	35.6 (9.7)	35.9 (8.3)	39.0 (8.3)	35.5 (8.8)
Mean # OUD Criteria (SD)	7.5 (2.2)	7.3 (2.6)	8.1 (2.2)	8.5 (2)	8.3 (2.1)	8.6 (2.1)	9.0 (1.4)	8.8 (1.5)	9.0 (1.4)
*n* families/persons ^c^	42/91	21/42	2/4	70/142	20/40	9/20	0/0	0/0	0/0

Abbreviation: OUD, opioid use disorder; SD, standard deviation. a. Not ceased <6 months; ceased >1 year; not classified: 6 months < time since last use <1 year; b. not ceased <1 year; ceased >1 year; not classified: time since last use = 1 year; c. persons is the total number of persons in all families.

**Table 2:** **Top-ranked** genome-wide association study (GWAS) findings for opioid cessation (*p* < 1 × 10^−5^) in African American and European ancestry participants, and in the combined sample.

Chr:position	ID	MA	Locus	African American					European Ancestry					Total	
				MAF	OR	N	*p*	Dir	MAF	OR	N	*p*	Dir	OR	*P*
7:55887138	rs11767417	T	*SEPT14*	0.31	1.29	1130	1.06 × 10^−2^	+++	0.11	1.50	2919	2.02 × 10^−5^	++-+	1.39	1.30 × 10^−6^
9:9783693	rs4740988	T	*PTPRD*	0.24	1.48	1068	4.33 × 10^−4^	++?	0.25	1.25	2919	6.62 × 10^−4^	++++	1.31	2.24 × 10^−6^
2:76417353	rs10209663	C	*SUCLA2P2/AC073091.2*	0.40	0.69	1130	2.20 × 10^−4^	−−−	0.09	0.73	2919	4.11 × 10^−3^	−−−−	0.71	3.09 × 10^−6^
12:19767596	rs10743328	C	*AEBP2*	0.13	1.36	1130	7.53 × 10^−3^	+++	0.12	1.50	2265	1.41 × 10^−4^	+?++	1.43	3.37 × 10^−6^
8:2082245	rs36098404	G	*MYOM2*	0.26	0.93	1130	4.53 × 10^−1^	+−−	0.07	1.72	2919	4.11 × 10^−6^	++++	1.22	7.77 × 10^−2^
12:81223948	rs12316031	T	*MIR617*	0.28	1.43	1068	5.37 × 10^−3^	++?	0.10	1.48	2919	2.88 × 10^−4^	++++	1.46	5.84 × 10^−6^
20:10086110	rs592026	G	*SNAP25-AS1*	0.11	1.11	822	5.48 × 10^−1^	+??	0.19	1.44	2919	6.53 × 10^−6^	+−++	1.24	3.23 × 10^−3^
8:121847800	rs4367595	A	*RP11-713M15.2/RP11-369K17.1*	0.22	1.27	1130	4.76 × 10^−2^	++−	0.37	1.27	2919	7.45 × 10^−5^	++++	1.27	9.47 × 10^−6^
18:40805792	rs114592700	C	*RIT2-SYT4*	0.05	1.28	822	3.03 × 10^−1^	+??	0.07	1.74	2919	9.50 × 10^−6^	++++	1.61	3.22 × 10^−4^

Abbreviations: Chr: position, chromosome and map position (in base pairs); MA, minor allele; MAF, minor allele frequency; Dir, effect direction in the OMNI, HCE, and MEGA array samples in the Yale–Penn dataset and the CATS dataset, respectively.

**Table 3:** Association of and model fit between polygenic risk scores for substance use disorders and opioid-related traits in the UK Biobank with opioid cessation.

Predictor Trait	GWAS *n*	GWAS *p* threshold	PRS *p*_adj_	R^2^
Former smoker	83,133	1	4.44 × 10^−3^	0.0075
Former drinker	21,894	0.092	6.24 × 10^−4^	0.0099
Back pain for >3 months	84,489	0.18	1.25 × 10^−7^	0.021

Abbreviations: GWAS *n*, sample size of the GWAS for the trait from which summary statistics for the associated variants were obtained; GWAS *p* threshold, *p*-value threshold for including a variant in polygenic risk score (PRS) construction; PRS *p*_adj_, adjusted *p*-value for the trait PRS; R^2^, variance in opioid cessation explained by the trait PRS.

## Supplementary Materials

The following are available online at https://www.mdpi.com/2077-0383/9/1/180/s1, Figure S1: Derivation of opioid cessation study subjects from the Yale–Penn dataset; Figure S2: QQ plot of opioid cessation meta-analysis results; Figure S3: Manhattan plot of opioid cessation meta-analysis; Figure S4: Plots showing association, linkage disequilibrium (LD), and recombination rates in regions of four biologically relevant loci yielding suggestive evidence of association (*p* < 1 × 10^−5^); Table S1: Genes in significantly enriched pathways from MAGENTA in European Americans.
