# Supplementary material for: Genome-Wide Association Study of Opioid Cessation
Source: J Clin Med. 2020 Jan 9;9(1):180. doi: 10.3390/jcm9010180 (PMC7019731; doi:10.3390/jcm9010180)
Supplement: Supplementary file 1 [file jcm-09-00180-s001.pdf]

Table S1. Genes in significantly enriched pathways derived from GWAS results in the European ancestry sample.

(A) Vitamin D Metabolism and Pathway

Gene.	Chr	Start Pos.	End Pos.	Size (kb)	Number of SNPs	Number of independent SNPs	Gene p-value	Top-ranked SNP			
								ID	Z-score	*p*-Value	Odds Ratio
**COX15**	**10**	**101468504**	**101492423**	**24**	**197**	**5**	1.14×10^{-1}	rs11190244	2.59	9.63×10^{-3}	0.85
CYP2D6	22	42522500	42526883	4	217	8	2.18×10^{-1}	rs742086	2.33	1.98×10^{-2}	1.18
CYP24A1	20	52769987	52790516	21	261	28	2.02×10^{-1}	rs1298513	2.88	3.92×10^{-3}	0.84
CYP27B1	12	58156116	58160976	5	79	7	1.07×10^{-1}	rs182005106	2.37	1.79×10^{-2}	1.29
POR	7	75544419	75616173	72	238	13	2.35×10^{-2}	rs11311523	3.16	1.56×10^{-3}	1.23
RARA	17	38465422	38513895	48	15	11	1.30×10^{-1}	rs59214602	2.25	2.42×10^{-2}	1.20
RARB	3	25469753	25639422	170	607	56	2.49×10^{-1}	rs17016192	2.87	3.77×10^{-3}	0.72
RARG	12	53604352	53626036	22	65	11	1.23×10^{-1}	rs73309166	2.44	1.48×10^{-2}	1.36

(B) FGF Signaling Pathway

Gene	Chr	Start Pos.	End Pos.	Size (kb)	Number of SNPs	Number of independent SNPs	Gene *p*-Value	Top-ranked SNP			
								ID	Z-score	*p*-Value	Odds Ratio
**FGF2**	**4**	**123747862**	**123819390**	**72**	**255**	**15**	1.05×10^{-3}	rs35287534	3.77	1.62×10^{-4}	0.76
FGF3	11	69624735	69634192	9	203	33	1.15×10^{-1}	rs10908251	2.89	3.89×10^{-3}	0.78
FGF4	11	69587796	69590171	2	249	36	1.66×10^{-1}	rs10908251	2.89	3.89×10^{-3}	0.78
FGF5	4	81187741	81212171	24	279	21	1.72×10^{-1}	rs3796594	2.74	6.06×10^{-3}	0.74
FGF6	12	4543307	4554780	11	177	19	1.37×10^{-1}	rs2244388	2.76	5.84×10^{-3}	0.83
FGF7	15	49715374	49779523	64	361	10	1.74×10^{-1}	rs1583060	2.62	8.93×10^{-3}	1.34
FGF12	3	191857181	192445388	588	887	71	1.76×10^{-1}	rs2221465	3.17	1.52×10^{-3}	1.24
FGFR1	8	38268655	38326352	58	205	16	1.10×10^{-1}	rs10637920	2.67	7.03×10^{-3}	1.24
FGFR3	4	1795038	1810599	16	103	7	6.72×10^{-2}	rs712983	2.65	8.13×10^{-3}	0.82
FGFR2	10	123237843	123357972	120	366	42	5.64×10^{-2}	rs10886938	3.20	1.38×10^{-3}	0.79
PPP2R1B	11	111597631	111637169	40	89	5	1.41×10^{-1}	rs61899413	2.27	2.34×10^{-2}	1.21
PPP2R2C	4	6322304	6474326	152	416	56	2.30×10^{-1}	rs113823191	2.87	4.06×10^{-3}	0.72
PPP2R5A	1	212458878	212535205	76	371	21	2.30×10^{-1}	rs11405845	2.57	1.02×10^{-2}	1.20
PPP2R5B	11	64692142	64701950	10	40	12	2.02×10^{-1}	rs56339918	2.24	2.55×10^{-2}	1.15
PPP2R5D	6	42952329	42980080	28	257	7	4.83×10^{-2}	rs3805946	2.94	3.30×10^{-3}	1.19
MAP3K6	1	27681669	27693337	12	79	10	8.97×10^{-2}	rs4246507	2.56	1.05×10^{-2}	1.19
SPRY1	4	124317955	124324909	7	189	24	1.38×10^{-2}	rs300576	3.35	8.03×10^{-4}	1.37
SPRY2	13	80910111	80915086	5	129	11	8.17×10^{-2}	rs9601380	2.68	7.29×10^{-3}	0.82
MRPL38	17	73894723	73901181	6	199	11	1.18×10^{-1}	rs142057056	2.67	7.66×10^{-3}	1.50
PEBP4	8	22570764	22785421	215	366	57	9.68×10^{-2}	rs200317753	3.05	2.27×10^{-3}	0.76

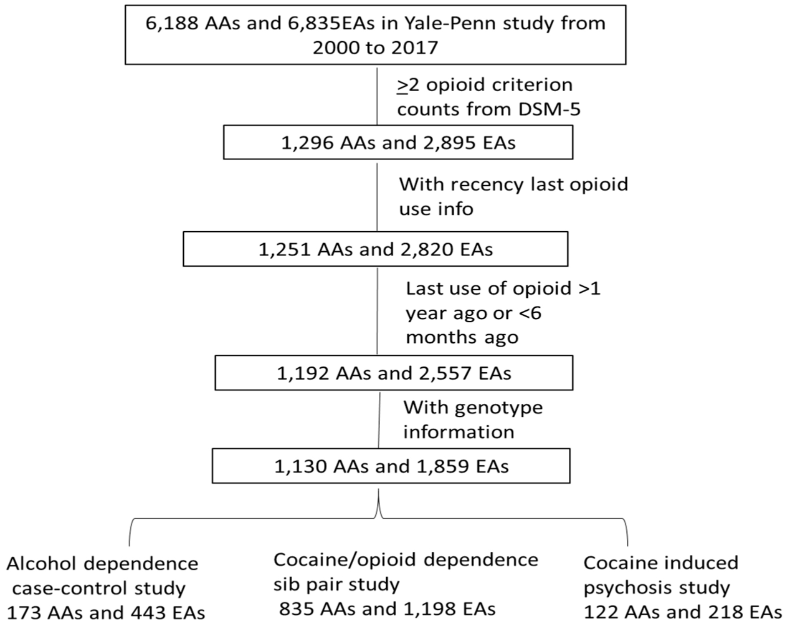

Figure S1. Derivation of opioid cessation study subjects from the Yale-Penn dataset.

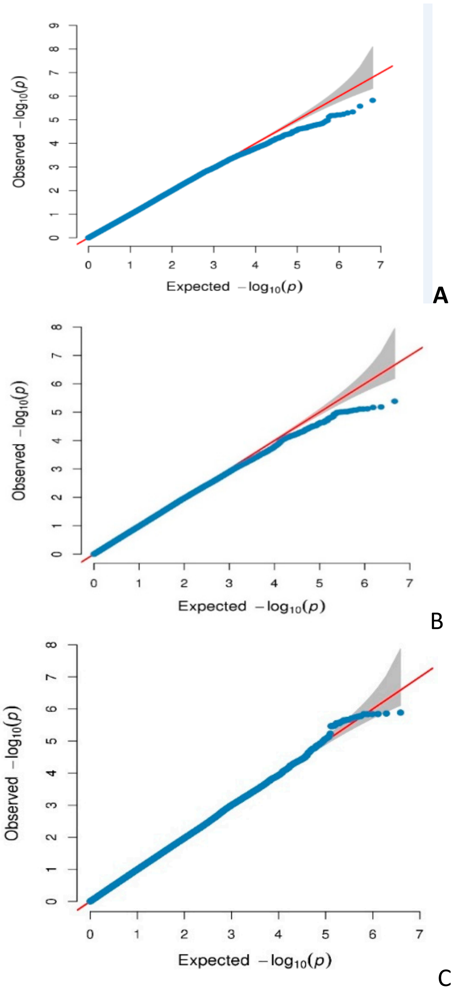

Figure S2. QQ plot of opioid cessation meta-analysis results for (**A**) African American (AAs) in the Yale-Penn dataset, (**B**) European Americans (EAs) in the Yale-Penn + CATS datasets, and (**C**) AAs and EAs combined. The genomic inflation factors are 0.985 for AAs, 0.981 for EAs, and 0.991 for the total sample.

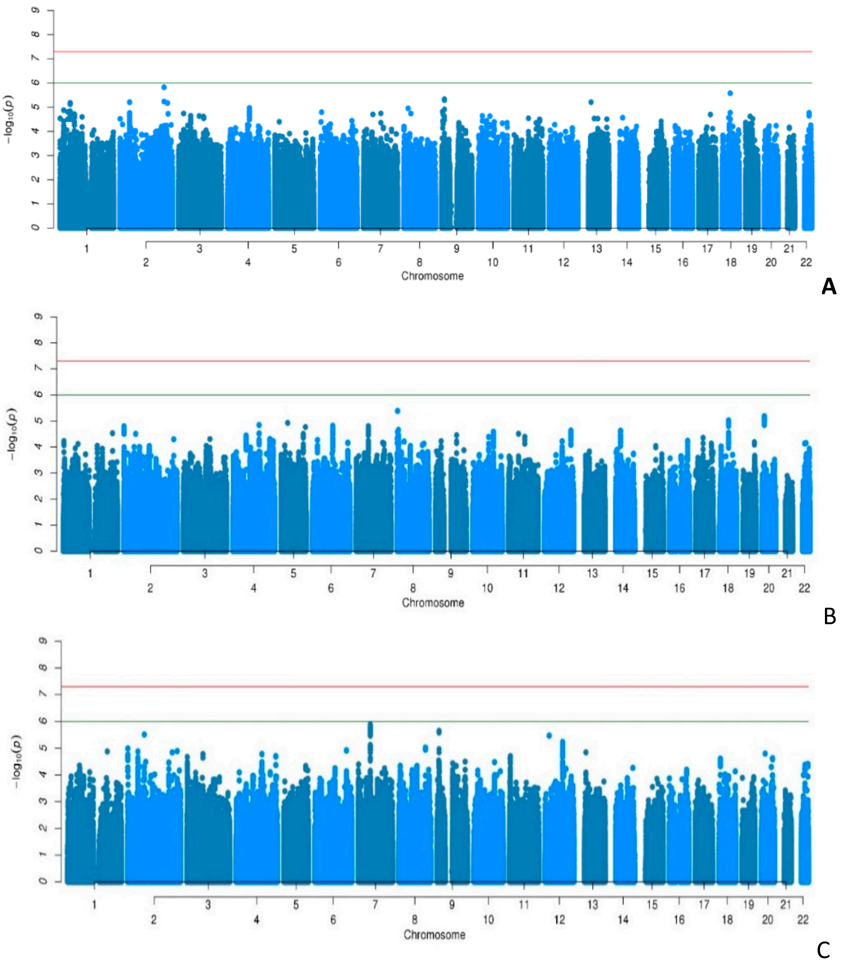

Figure S3. Manhattan plots for opioid cessation GWAs results IN (**A**) African American (AA) participants in the Yale-Penn dataset, (**B**) European ancestry (EA) participants in the Yale-Penn +CATS datasets, and (**C**) AAs and EAs combined. Red line indicates genome-wide significance threshold (p = 5 × 10^{-8}), green line indicates sub genome-wide significance threshold (p = 1 × 10^{-6}).

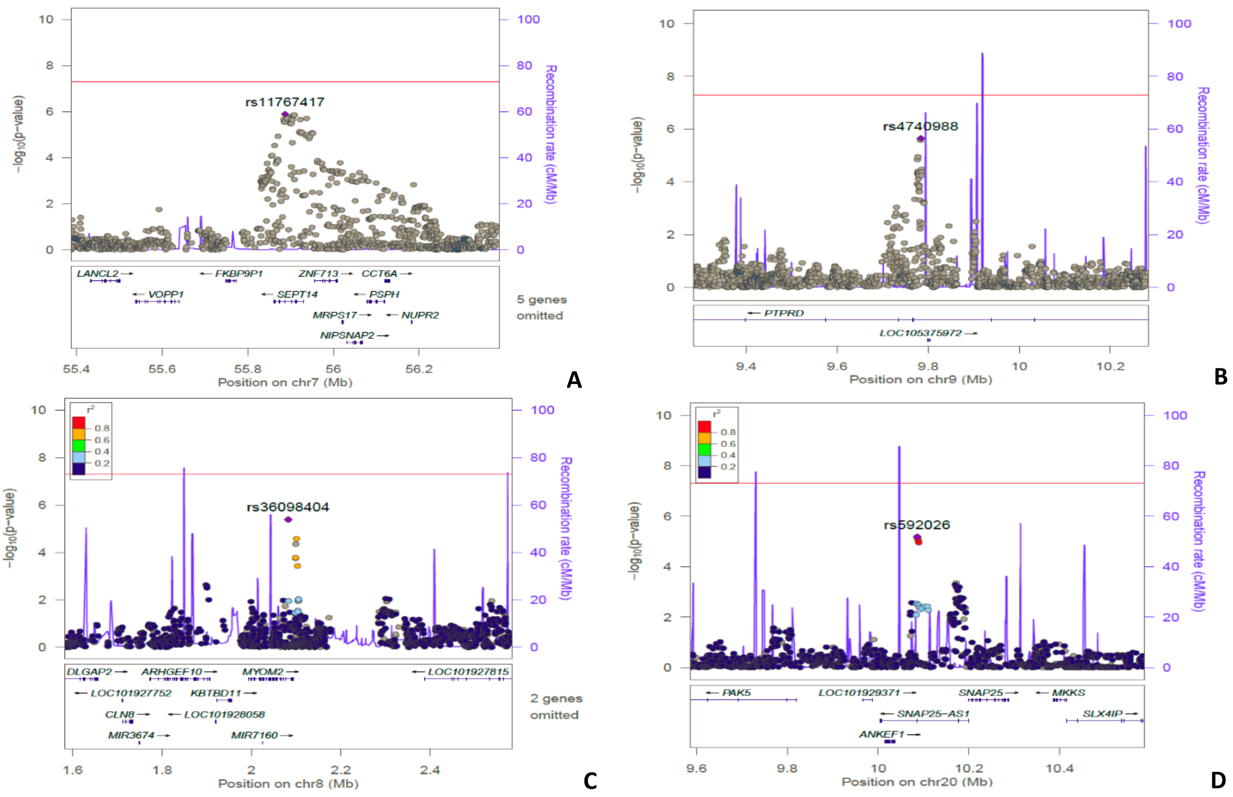

Figure S4. Association, linkage disequilibrium (LD) and recombination rates in regions of four biologically relevant loci yielding suggestive evidence of association ($p < 1{\times}10^{-5}$) in the combined European ancestry (EA) and African American (AA) datasets (**panels A and B**) or EA datasets only (**panels C and D**). Red line indicates the genome-wide significance threshold. Degree of LD between the top variant and other variants in the non-admixed samples is indicated by color according to the key in the top left corner. Recombination rates are indicated by purple vertical lines. Arrows beside gene symbols indicate the direction of transcription. Rectangles in the gene structures shown beneath each symbol are exons.
